# High-throughput prediction of eucalypt lignin syringyl/guaiacyl content using multivariate analysis: a comparison between mid-infrared, near-infrared, and Raman spectroscopies for model development

**DOI:** 10.1186/1754-6834-7-93

**Published:** 2014-06-17

**Authors:** Jason S Lupoi, Seema Singh, Mark Davis, David J Lee, Merv Shepherd, Blake A Simmons, Robert J Henry

**Affiliations:** 1 Queensland Alliance for Agriculture and Food Innovation, University of Queensland, 306 Carmody Road, St. Lucia, QLD 4072, Australia; 2 Joint BioEnergy Institute, Lawrence Berkeley National Laboratory, 5885 Hollis Street, Emeryville, CA 94608, USA; 3 Biological and Materials Science Center, Sandia National Laboratories, 7011 East Avenue, Livermore, CA 94551, USA; 4 BioEnergy Science Center, Oak Ridge National Laboratory, 1 Bethel Valley Rd, Oak Ridge, TN 37831, USA; 5 National Bioenergy Center, National Renewable Energy Laboratory, 15013 Denver West Parkway, Golden, CO 80401, USA; 6 Forest Industries Research Centre, University of the Sunshine Coast and Queensland Department of Agriculture, Fisheries and Forestry, Locked Bag 4, Maroochydore DC, QLD 4558, Australia; 7 Southern Cross Plant Science, Southern Cross University, Military Road, East Lismore, NSW 2480, Australia

**Keywords:** Biomass, Raman spectroscopy, Near-infrared spectroscopy, Fourier-transform infrared spectroscopy, High-throughput, Multivariate analysis, Lignin S/G

## Abstract

**Background:**

In order to rapidly and efficiently screen potential biofuel feedstock candidates for quintessential traits, robust high-throughput analytical techniques must be developed and honed. The traditional methods of measuring lignin syringyl/guaiacyl (S/G) ratio can be laborious, involve hazardous reagents, and/or be destructive. Vibrational spectroscopy can furnish high-throughput instrumentation without the limitations of the traditional techniques. Spectral data from mid-infrared, near-infrared, and Raman spectroscopies was combined with S/G ratios, obtained using pyrolysis molecular beam mass spectrometry, from 245 different eucalypt and *Acacia* trees across 17 species. Iterations of spectral processing allowed the assembly of robust predictive models using partial least squares (PLS).

**Results:**

The PLS models were rigorously evaluated using three different randomly generated calibration and validation sets for each spectral processing approach. Root mean standard errors of prediction for validation sets were lowest for models comprised of Raman (0.13 to 0.16) and mid-infrared (0.13 to 0.15) spectral data, while near-infrared spectroscopy led to more erroneous predictions (0.18 to 0.21). Correlation coefficients (r) for the validation sets followed a similar pattern: Raman (0.89 to 0.91), mid-infrared (0.87 to 0.91), and near-infrared (0.79 to 0.82). These statistics signify that Raman and mid-infrared spectroscopy led to the most accurate predictions of S/G ratio in a diverse consortium of feedstocks.

**Conclusion:**

Eucalypts present an attractive option for biofuel and biochemical production. Given the assortment of over 900 different species of *Eucalyptus* and *Corymbia*, in addition to various species of *Acacia*, it is necessary to isolate those possessing ideal biofuel traits. This research has demonstrated the validity of vibrational spectroscopy to efficiently partition different potential biofuel feedstocks according to lignin S/G ratio, significantly reducing experiment and analysis time and expense while providing non-destructive, accurate, global, predictive models encompassing a diverse array of feedstocks.

## Background

Second-generation biofuels from lignocellulosic biomass have been progressively explored as plausible pathways to relinquishing global dependency on greenhouse gas-emitting fossil fuels [1-3]. Given the multitude of possible plants, the development of high-throughput analytical techniques capable of screening large arrays of feedstocks is paramount to isolating ideal candidates for biofuel and biochemical research and production. The measurement of biomass phenotypic parameters such as chemical composition, enzymatic hydrolysis sugar release, and the ratio of syringyl (S)-to-guaiacyl (G) lignin moieties can aid in identifying biomass species possessing traits found to play important roles in diminishing biomass recalcitrance. The effects different feedstock traits have on cell wall deconstruction are multifaceted, and no one superlative characteristic has been identified. Therefore, the collective high-throughput measurements of various traits can rapidly illuminate plants of interest. For example, Sykes *et al.* used high-throughput pyrolysis molecular beam mass spectrometry (pyMBMS) to screen approximately 800 poplar trees based on lignin content and S/G ratio [4].

Lignin is a three-dimensional, structurally complex, biopolymer comprised of phenylpropanoid units, designated as S, G, and p-coumaryl (H) components [5,6]. The ratio of S- to G-lignin provides a pivotal parameter for gauging the expected chemical reactivity of delignifying plant cell walls and for determining the energy requirements for pulping and bleaching feedstocks [7-9]. The correlation of S/G ratios to monomeric sugar release following hydrolysis of biomass has been explored, revealing conflicting results [7,10,11]. A slight decrease in the S/G ratio of hybrid poplar was shown to improve the rate of dilute acid hydrolysis [7]. While counter to the authors’ original hypothesis, the results demonstrate the applicability of using S/G ratios as an indicator of monomeric sugar yield. When juxtaposed with other correlative studies of S/G ratio and saccharification yield, high S/G ratios have occasionally resulted in the release of larger quantities of monomeric sugars [10,11]. Thus, although the exact effect of lignin S/G ratio on enzymatic hydrolysis of polysaccharides has not been elucidated, this metric has proven to be integral to understanding the role lignin structure plays in deconstructing biomass.

The techniques traditionally employed to determine lignin S/G ratios include wet chemistry methods like nitrobenzene oxidation or thioacidolysis, and instrumental approaches such as nuclear magnetic resonance, gas chromatography/mass spectrometry (GC/MS), pyrolysis GC/MS (pyGCMS), and pyMBMS [12]. These methods can be expensive, labor-intensive, time-consuming, destructive, and/or utilize toxic reagents such as boron trifluoride etherate.

Vibrational spectroscopy, including Raman, near-infrared (NIR), and mid-infrared (MIR) has been shown to provide non-destructive, high-throughput, qualitative and quantitative assessments of lignin S/G ratios (Table 1) [13-26]. Typically, the spectral data are combined with analytical results from a traditional technique (pyrolysis/mass spectrometry, thioacidolysis/gas chromatography) for a subset of the samples, and the use of multivariate analysis, including principal component analysis (PCA) and partial least squares regression (PLS) allows the formation of robust models capable of classifying and predicting the analytes for the remaining samples. While many options have been explored in the search for robust, high-throughput analytical techniques to efficiently screen biomass via multivariate modeling, attempts to compare one lab’s results with another can be enervating due to the lack of standardization in model construction and in the statistics reported for model critique (Table 1). In order to accurately showcase the predictive power of these instrumental methods, it is imperative for forthcoming literature to include the statistics resultant from the rigorous validation of multivariate models.

**Table 1:** Evaluation of literature multivariate models for lignin S/G prediction

**Method**	**# of samples**	**SEC**^**1**^	**SEP**^**2**^	**RM-SEC**^**3**^	**RM- SECV**^**4**^	**Random Val. Set**	**RM-SEP**^**5**^	**r-Val**^**6**^	***R***^**2**^**Cal**^**7**^	***R***^**2 **^**Val**^**8**^	**F**^**9**^	**Ref**
**Raman**	10	-	-	0.02 (S) 0.04 (G)	-	N	0.04 (S) 0.04 (G)	*0.993 (S) 0.992 (G)*	0.996 (S) 0.997 (G)	0.985 (S) 0.986 (G)	-	[15]
**Raman**	55 (Cal) 25 (Val)	0.07	0.32	-	-	Y	-	0.935	*0.996*	*0.874*	5	[16]
**Raman**	63 (Cal) 30 (Val)	0.32	0.35	-	-	Y	-	0.919	*0.887*	*0.845*	2	[23]
**Raman**	9	-	-	-	-	N	-	-	-	0.983	-	[20]
**MIR**	5	-	-	-	-	-	-	-	-	-	-	[14]
**MIR**	5	-	-	-	-	-	-	-	-	-	-	[24]
**MIR**	65	-	-	-	-	-	-	-	-	0.91-0.98	-	[25]
**MIR**	15	-	-	-	-	-	-	-	-	-	-	[26]
**NIR**	42 (Cal) 36 (Val)	-	-	-	0.025-0.033	Y	0.025-0.036	*0.959-0.980*	0.95-0.97	0.92-0.96	3-5	[13]
**NIR**	267 (Cal)	-	-	0.176 (S) 0.202 (G) 0.005 (H)	0.201 (S) 0.202 (G) 0.005 (H)	N	-	0.979 (S) 0.979 (G) 0.843 (H)	0.968 (S) 0.957 (G) 0.731 (H)	0.958 (S) 0.957 (G) 0.710 (H)	8	[17]
**NIR**	135 (Cal) 45 (Val)	-	0.124	-	0.121	Y	-	*0.686*	0.583	0.47	5-7	[19]
**NIR**	26 (Cal) 8 (Val)	0.26	0.3	-	-	Y	-	*0.938*	0.96	0.88	6	[22]

^1^Standard Error of Calibration.

^2^Standard Error of Prediction.

^3^Root Mean Standard Error of Calibration.

^4^Root Mean Standard Error of Cross-Validation.

^5^Root Mean Standard Error of Prediction.

^6^Coefficient of Correlation for Validation Set.

^7^Coefficient of Determination for Calibration Set.

^8^Coefficient of Determination for Validation Set.

^9^#of Factors.

Italicized values calculated by the authors.

**MIR = mid-infrared spectroscopy, NIR = near-infrared spectroscopy, Cal = calibration set, Val = validation set, S = syringyl, G = guaiacyl, H =** ***p*-hydroxyphenol, Y = yes, N = no.**

Raman spectroscopy measures the amount of photon scattering from a molecule when irradiated with an excitation source, such as a laser [27]. The peaks in a Raman spectrum are characteristic of specific chemical bond vibrational modes, allowing qualitative structural assessments of the analyte as well as quantitation using peak heights and areas. Sample preparation is undemanding, the methodology does not destroy samples, and analytes in aqueous and complex matrices can routinely be evaluated, making Raman spectroscopy a robust, versatile, analytical tool. The use of Raman spectroscopy for the assembly of multivariate models capable of predicting biofuel traits has not been systematically explored, although recent instrumental advances such as the robust, hand-held, field-portable devices, make Raman spectroscopy an attractive option for further application for biomass analysis [28,29].

Fourier-transform (FT) Raman spectroscopy coupled with thioacidolysis has been used to develop PLS models for predicting lignin S/G ratios [16]. Fifty-five *Eucalyptus globulus* and *E. camaldulensis* samples were used to create a calibration matrix, while 25 randomly selected samples comprised the prediction set. The authors report a correlation coefficient (r) of 0.935 and a standard error of prediction (SEP) of 0.32 using a five factor model. In another study, assessing five different *Eucalyptus* species, predictive models of lignin S/G ratio using FT-Raman and PLS revealed slightly less accurate models (r = 0.919, SEP = 0.35), perhaps due to the inclusion of a wider variety of feedstocks [23]. However, due to the insertion of these other species, the authors were able to successfully build a more diverse model, capable of predicting traits from both heart- and sapwood, and differences in tree age.

FT-Raman spectral deconvolution between 1220 and 1530 cm^−1^ has been employed to determine lignin S/G ratios in nine feedstocks including *Arabidopsis thaliana* and *E. globulus*[20]. The authors developed a calibration equation to compare results obtained via pyGCMS with good correlation (coefficient of determination*, R*^*2*^ = 0.983), despite observed overlap between the spectral regions designated as S, G, or H lignin signatures, and vibrational modes due to polysaccharides cellulose and xylan. Near-infrared, dispersive, multichannel Raman spectroscopy coupled with thioacidolysis/GCMS data allowed the quantitation of S and G lignin content in 10 feedstocks, with emphasis on herbaceous plants [15]. A principal component regression model was generated that accurately predicted the S/G ratio for the majority of plants studied (*R*^*2*^ = 0.985-0.986, root mean standard error of prediction (RMSEP) = 0.02-0.04).

Mid-infrared (MIR) spectroscopy provides complementary information to Raman spectroscopy due to the difference in selection rules. In Raman spectroscopy, a change in the polarizability of an electron cloud is required for a molecule to be ‘active’, while in MIR spectroscopy, there must be a change in dipole [30]. Thus, the use of both techniques in tandem can present more complete structural assessments of analytes. The use of MIR spectroscopy to analyze biomass has been more frequently employed to obtain qualitative structural information rather than as a quantitative tool for development of multivariate predictive models, in part due to the strong absorption of water, making analysis of aqueous and biological samples difficult. Some recent endeavors employing MIR spectroscopy have explored quantifying lignin S/G ratios. del Rio *et al.* used MIR spectroscopy to develop a technique for estimating S/G ratios in lignin isolated from hemp, flax, jute, sisal, and abaca feedstocks [14]. Following the selected spectral processing of resolution enhancement, smoothing, and baseline correction, peak intensities at 1327 and 1271 cm^−1^ were used as a marker for S- and G-lignin, respectively. The calculated S/G ratios were found to agree with pyGCMS.

To date, the most widespread vibrational spectroscopy screening methods employ NIR spectroscopy. The straightforward instrumentation and sample handling in NIR spectroscopy has permitted the screening of large arrays of biomass feedstocks to identify those possessing the key traits necessary for efficient biofuel production including lignin S/G ratios [13,17,19,22]. Unlike Raman or MIR spectroscopies that characterize fundamental vibrational modes, NIR measures overtone and combination bands associated with C-H, N-H, O-H, and S-H moieties [31]. These spectral features are subtle and require spectral deconvolution or multivariate analysis for the elucidation of useful parameters.

Thioacidolysis/GCMS results were coupled with NIR spectra to develop a PLS model for predicting S/G ratios in transgenic aspen [22]. The authors report a calibration *R*^2^ of 0.96 and a standard error of calibration (SEC) of 0.26 using six factors and 26 spectra. Eight spectra were used to validate the PLS model, resulting in a *R*^2^ of 0.88, and SEP of 0.3. NIR spectra of 267 wild and transgenic poplar samples were also conjoined with S, G, and H lignin fractions measured using a streamlined, higher-throughput thioacidolysis protocol [17]. The resultant PLS model used 8 factors to explain the variance and a full cross-validation to determine the predictive capacity. The authors report *R*^2^ values 0.958 (S), 0.957 (G), and 0.710 (H) for model prediction and cross-validation RMSE values of 0.201 (S), 0.202 (G), and 0.005 (H). A randomly selected validation set was not employed to more rigorously test the model’s validity. Finally, NIR spectra and analytical pyrolysis were used to build PLS models to predict the S/G ratios of *E. globulus* trees [13]. The authors randomly selected 42 of 78 samples to compose the calibration model, using the remaining 36 samples for validation. A full cross-validation indicated that 3 to 5 factors were sufficient to explain the variance in the data, depending on the type of spectral processing utilized. The prediction *R*^2^ values ranged from 0.92 to 0.96 while the RMSEP values were between 0.024 and 0.036.

The nomination of appropriate second-generation biofuel candidates is paramount to reducing consumption of non-renewable energy sources [1,32,33]. Eucalypts possess attractive characteristics that increase their candidacy for further biofuel production studies, including the ability to grow on marginal land, in low nutrient and degraded soil, their proficiency of growing rapidly and in a wide variety of climates, the coppicing of many eucalypt species preventing the need to replant the tree, and the well-known understanding of breeding, growing, processing, and chemical composition of the biomass [34].

This manuscript describes the marriage of multivariate analysis, vibrational spectroscopy, and pyMBMS data to assemble PLS models capable of partitioning diverse eucalypts and *Acacias* according to lignin S/G ratio. MIR, NIR, and Raman spectroscopic methods are illustrated, including spectral acquisition and processing, and the resultant models are critiqued statistically in order to elect which technique(s) will provide the most accurate prediction of lignin S/G ratios. It is demonstrated that MIR and Raman spectroscopy, although sporadically portrayed in current literature, are capable of providing powerful high-throughput analytical tools for lignin S/G analysis. Additionally, while many previous studies have focused on one type of biomass (*E. globulus*, or transgenic and wild-type poplar), the global models constructed in this study incorporate three genera encompassing 17 different species of biomass, including wild-type and hybrid *Corymbia*, which, to the authors’ knowledge, have not been extensively characterized.

## Results and discussion

### Compilation of reference set

The reference set of wood samples selected for pyMBMS analysis was designed to represent the greatest amount of variance between the diverse collections of plants. This was achieved by performing a PCA of the MIR and NIR spectral data. In PCA, samples are classified according to similarity, thus when considering a scores plot when two samples have neighboring coordinates they are more similar; the farther they are from each other, the more dissimilar. A Hotelling T^2^ ellipse can be drawn around the samples to illustrate which samples lay farthest from the mean sample. The samples that fell outside or near the periphery of the ellipse were designated as ‘unique’ samples for inclusion in the reference data set, as capturing this variance in a predictive model would most likely encompass future samples (Additional file 1: Figure S1). The remainder of the reference set was comprised of randomly selected plants that lay inside of the Hotelling T^2^ ellipse and were more representative of the sample majority.

### Spectral data acquisition

**Figure 1:**
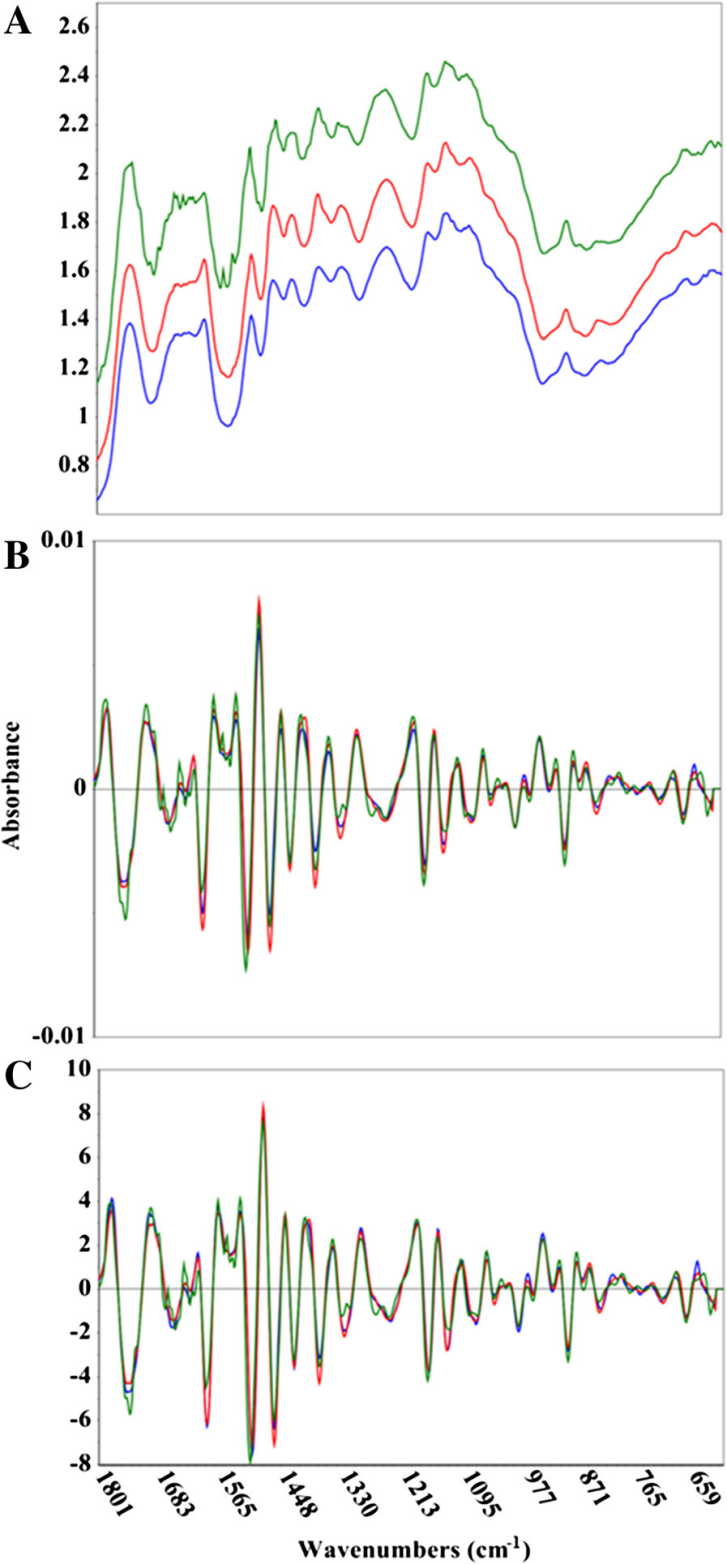
**Comparison of raw and pretreated mid-infrared spectral data.** Mid-infrared spectra of *Acacia microbotrya* (green), *Corymbia* hybrid (blue), and *Eucalyptus globulus* subspecies *maidenii* (red). The upper panel **(A)** shows the untreated spectral data, while the middle **(B)** and bottom **(C)** panels show the second derivative, and second derivative + standard normal variate (SNV) spectral transformations, respectively. The x-axis is in wavenumbers while the y-axis is the absorbance.

**Figure 2:**
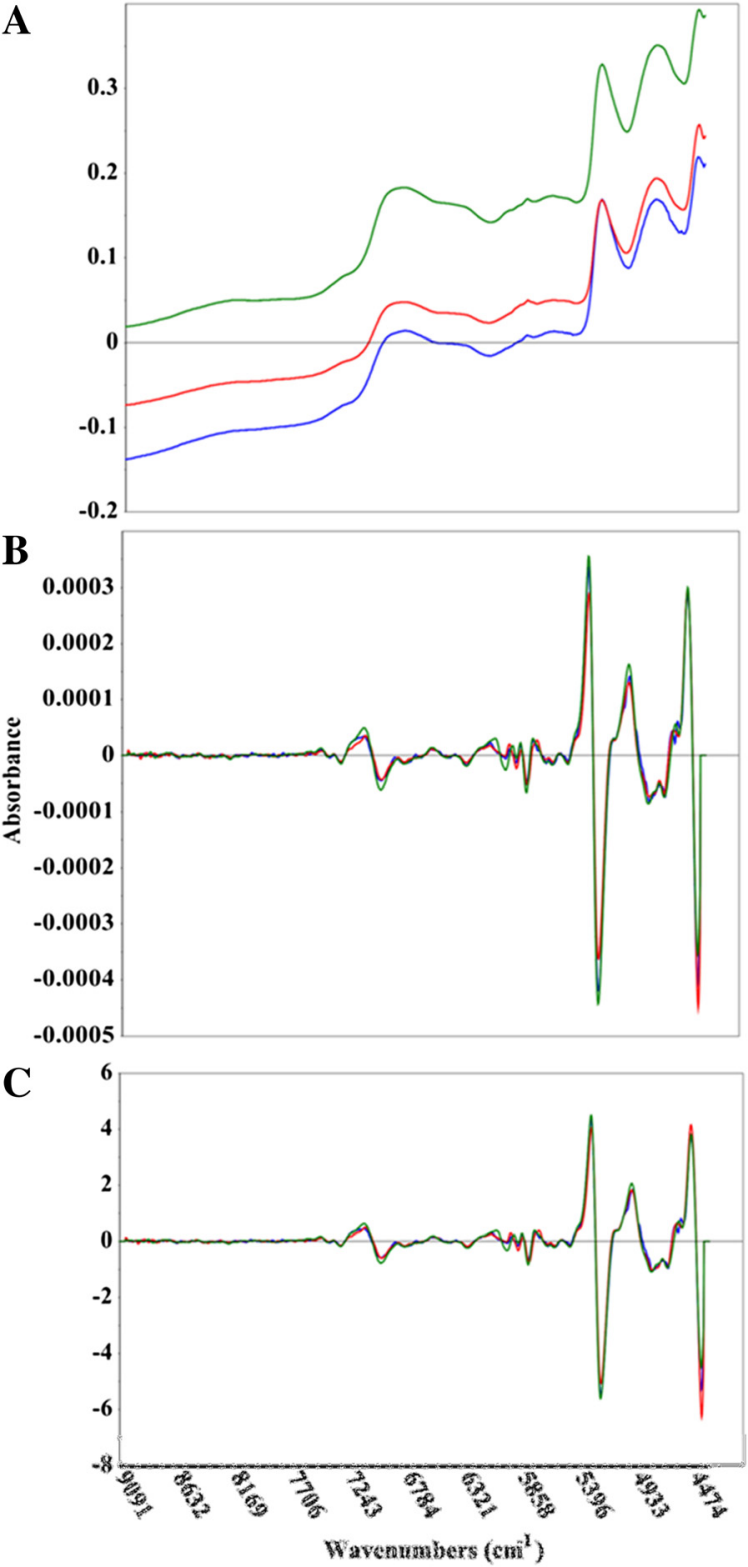
**Comparison of raw and pretreated near-infrared spectral data.** Near-infrared spectra of *Acacia microbotrya* (green), *Corymbia* hybrid (blue), and *Eucalyptus globulus* subspecies *maidenii* (red). The upper panel **(A)** shows the untreated spectral data, while the middle **(B)** and bottom **(C)** panels show the second derivative, and second derivative + standard normal variate (SNV) spectral transformations, respectively. The x-axis is in wavenumbers while the y-axis is the absorbance.

**Figure 3:**
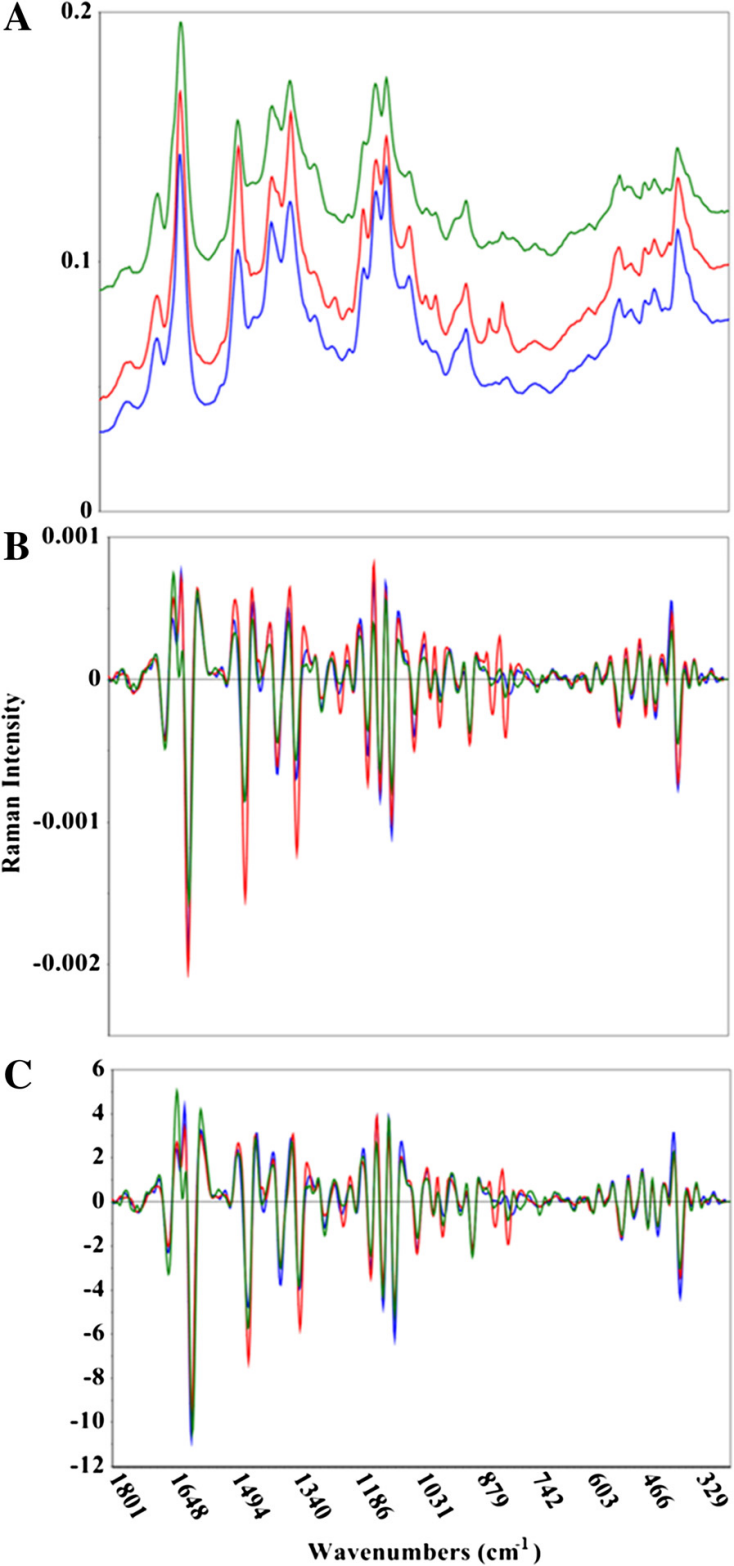
**Comparison of raw and pretreated Raman spectral data.** Raman spectra of *Acacia microbotrya* (green), *Corymbia* hybrid (blue), and *Eucalyptus globulus* subspecies *maidenii* (red). The upper panel **(A)** shows the untreated spectral data, while the middle **(B)** and bottom **(C)** panels show the second derivative, and second derivative + standard normal variate (SNV) spectral transformations, respectively. The x-axis is in wavenumbers, while the y-axis shows the Raman intensity.

MIR, NIR, and Raman spectra were collected for 245 diverse eucalypt and *Acacia* trees in 96-well plates. Figures 1, 2, and 3 provide a spectral comparison between the three analytical methods. The spectral acquisition parameters (see Methods) were optimized to maximize the signal-to-noise (S/N). The tunable nature of the Raman excitation source is one of the main advantages to using Raman spectroscopy. It allows a two-pronged approach to S/N optimization by amplifying the signal via higher excitation powers (although the noise can also increase), and decreasing the noise by augmenting the number of spectral scans. With the MIR and NIR instruments, the sole way to increase S/N is by performing more scans of the data, which only reduces the noise. By intensifying the laser power, previously veiled spectral features can be elucidated, which ultimately can lead to more distinguished spectra. Multivariate models flourish when there is significant variance in the data set.

### Multivariate model assembly and evaluation

The lignin S/G ratios quantified by pyMBMS (Additional file 1: Table S2) were linked with MIR, NIR, and Raman spectral data. The standard error of the laboratory (SEL) calculated for the pyMBMS reference data (0.05 to 0.06) is listed in Tables 2 and 3, as well as Additional file 1: Table S2. This value represents the lowest possible predictive error a model can contain as a model cannot have lower error than the data used in its construction. Iterations of spectral processing techniques allowed the selection of those treatments that led to the most robust PLS models (Table 2). First and second derivative transformations were employed to remove additive effects from the spectra, such as baseline offsets. Spectral smoothing was necessary to remove the additional spectral noise concomitant with performing derivative transformations. It is imperative that the spectra not be over-smoothed, as this can be deleterious to vital spectroscopic information. Multiplicative effects like variations in biomass particle size were removed using techniques such as standard normal variate (SNV), multiplicative scatter correction (MSC), or extended MSC (EMSC). Combinations of these spectral corrections were used to remove physical differences from the samples such that predictive models focused on chemical distinctions (second derivative + SNV).

**Table 2:** Comparison of PLS calibration models using vibrational spectroscopy and pyrolysis molecular beam mass spectrometry

**Method**	**SEL calibration**^**1,a**^	**RMSEC**^**2,a**^	**RM- SECV**^**3,a**^	***R***^**2 **^**Cal**^**4**^	***R***^**2 **^**CV**^**5**^	**F**^**6**^	**Outliers**^**7**^
**Raman 2**^**nd **^**deriv (19 pt) + SNV 32 scans**	0.05	0.13	0.14	0.83 ± 0.02	0.81 ± 0.02	4-5	2
**Raman 1**^**st **^**deriv (7 pt) + EMSC 32 scans**	0.05	0.13	0.14	0.845 ± 0.003	0.82 ± 0.01	4-5	3
**Raman EMSC + 2**^**nd **^**deriv (15 pt) 96 scans**	0.05	0.13	0.14	0.83 ± 0.01	0.81 ± 0.01	5-6	2-3
**Raman 2**^**nd **^**deriv (15 pt) + SNV 96 scans**	0.05	0.13	0.13	0.84 ± 0.01	0.82 ± 0.01	4-5	4-5
**MIR EMSC + 2**^**nd **^**deriv (15 pt)**	0.05	0.13	0.14	0.84 ± 0.03	0.81 ± 0.03	3-4	1-2
**MIR 2**^**nd **^**deriv (17 pt) + MSC**	0.05	0.13	0.14	0.82 ± 0.01	0.78 ± 0.01	3-4	1-2
**MIR 2**^**nd **^**deriv (17 pt) + SNV**	0.05	0.13	0.14	0.85 ± 0.02	0.82 ± 0.03	3-4	2-3
**NIR EMSC + 2**^**nd **^**deriv (25 pt)**	0.05	0.17	0.18	0.73 ± 0.01	0.681 ± 0.004	4-5	4-7
**NIR 2**^**nd **^**deriv (25 pt) + MSC**	0.05	0.17	0.18	0.72 ± 0.02	0.68 ± 0.02	4-6	1-5
**NIR 2**^**nd **^**deriv (25 pt) + SNV**	0.05	0.16	0.17	0.74 ± 0.01	0.70 ± 0.02	4-5	2-3

^1^Standard Error of the Laboratory for the calibration data.

^2^Root Mean Standard Error of Calibration.

^3^Root Mean Standard Error of Cross-Validation.

^4^Coefficient of determination for calibration set.

^5^Coefficient of determination for full cross-validation.

^6^Average number of factors used in model construction.

^7^Number of outliers removed from calibration models.

^a^Average errors of 3 randomly generated models using data provided. Models were not statistically different.

The numbers listed parenthetically reflect the degree of Savitzky-Golay spectral smoothing.

Statistical values are the average of 3 independent models.

MIR = mid-infrared spectroscopy, NIR = near-infrared spectroscopy, EMSC = extended multiplicative scatter correction, MSC = multiplicative scatter correction, SNV = standard normal variate.

**Table 3:** Comparison of PLS predictive models using vibrational spectroscopy and pyrolysis molecular beam mass spectrometry

**Method**	**SEL validation**^**1,a**^	**SEP**^**2,a**^	**RMSEP**^**3,a**^	**r-Val**^**4**^	***R***^**2 **^**Val**^**5**^	**Outliers**^**6**^
**Raman 2**^**nd **^**deriv (19 pt) + SNV 32 scans**	0.05	0.14	0.13	0.89 ± 0.04	0.79 ± 0.08	1
**Raman 1**^**st **^**deriv (7 pt) + EMSC 32 scans**	0.05	0.13	0.13	0.91 ± 0.02	0.83 ± 0.04	1
**Raman EMSC + 2**^**nd **^**deriv (15 pt) 96 scans**	0.05	0.14	0.15	0.90 ± 0.02	0.81 ± 0.04	0
**Raman 2**^**nd **^**deriv (15 pt) + SNV 96 scans**	0.06	0.17	0.16	0.86 ± 0.02	0.74 ± 0.04	0
**MIR EMSC + 2**^**nd **^**deriv (15 pt)**	0.05	0.14	0.13	0.87 ± 0.06	0.8 ± 0.1	1
**MIR 2**^**nd **^**deriv (17 pt) + MSC**	0.05	0.14	0.14	0.91 ± 0.01	0.83 ± 0.01	1
**MIR 2**^**nd **^**deriv (17 pt) + SNV**	0.05	0.15	0.15	0.87 ± 0.02	0.76 ± 0.03	1
**NIR EMSC + 2**^**nd **^**deriv (25 pt)**	0.06	0.19	0.20	0.79 ± 0.01	0.62 ± 0.01	0
**NIR 2**^**nd **^**deriv (25 pt) + MSC**	0.06	0.18	0.18	0.82 ± 0.04	0.67 ± 0.07	1
**NIR 2**^**nd **^**deriv (25 pt) + SNV**	0.06	0.22	0.21	0.80 ± 0.04	0.65 ± 0.07	1

^1^Standard Error of the Laboratory for the validation data.

^2^Standard Error of Prediction.

^3^Root Mean Standard Error Prediction.

^4^Correlation coefficient for the validation set.

^5^Pearson coefficient of determination for validation.

^6^Number of outliers removed from validation models.

^a^Average errors of three randomly generated models using data provided. Models were not statistically different.

The numbers listed parenthetically reflect the degree of Savitzky-Golay spectral smoothing.

Statistical values are the average of 3 independent models.

MIR = mid-infrared spectroscopy, NIR = near-infrared spectroscopy, EMSC = extended multiplicative scatter correction, MSC = multiplicative scatter correction, SNV = standard normal variate.

Tables 2 and 3 provide an assessment of the models .constructed using MIR, NIR, and Raman spectral data. The root mean standard error of cross-validation (RMSECV) is a measurement of the error in the predicted lignin S/G ratio when cross-validation is employed to assess the models. Cross-validation is also called the ‘leave-one-out’ method, as one sample from the data matrix is removed and subsequently predicted using the remaining samples. This technique proceeds for a set number of calibration samples (random CV) or is performed for all samples (full CV). While the RMSECV is a good indicator of a model’s accuracy, a more aggressive way of evaluating PLS models is by using a randomly produced validation set, and monitoring the resultant RMSEP. This reflects the error that can be expected in the model when true predictions of unknown variables are attempted.

Another metric that facilitates the development of robust multivariate models is the correlation coefficient (r) or the coefficient of determination (*R*^2^). These parameters reflect the accuracy of the linear experimental trend line versus the ideal. Again, while probing these values for CV to isolate which spectral processing techniques result in the most robust, accurate models, assessing the r and *R*^*2*^ magnitudes for the prediction of a true validation set permits a more rigorous analysis of model performance.

The number of factors used for model construction is also reported in Table 2. Over-fitting the data can result if more than the optimal number of factors is employed, as these superfluous factors attempt to explain random noise in the spectra. Adding and subtracting the number of factors used in the calculation of a model can diagnose over-fitting of the data. For example, using more factors than is optimal may result in seemingly more accurate calibration models, but will result in more erroneous validation and prediction. Evaluation of residual variance plots or Scree plots (Additional file 1: Figure S2) aided in determining the appropriate number of factors to use for the model. Another technique to assess over-fitting is by gauging the validation metrics, namely the *R*^*2*^ and RMSEP as these parameters will be significantly less accurate from those obtained using cross-validation if over-fitting has occurred.

**Figure 4:**
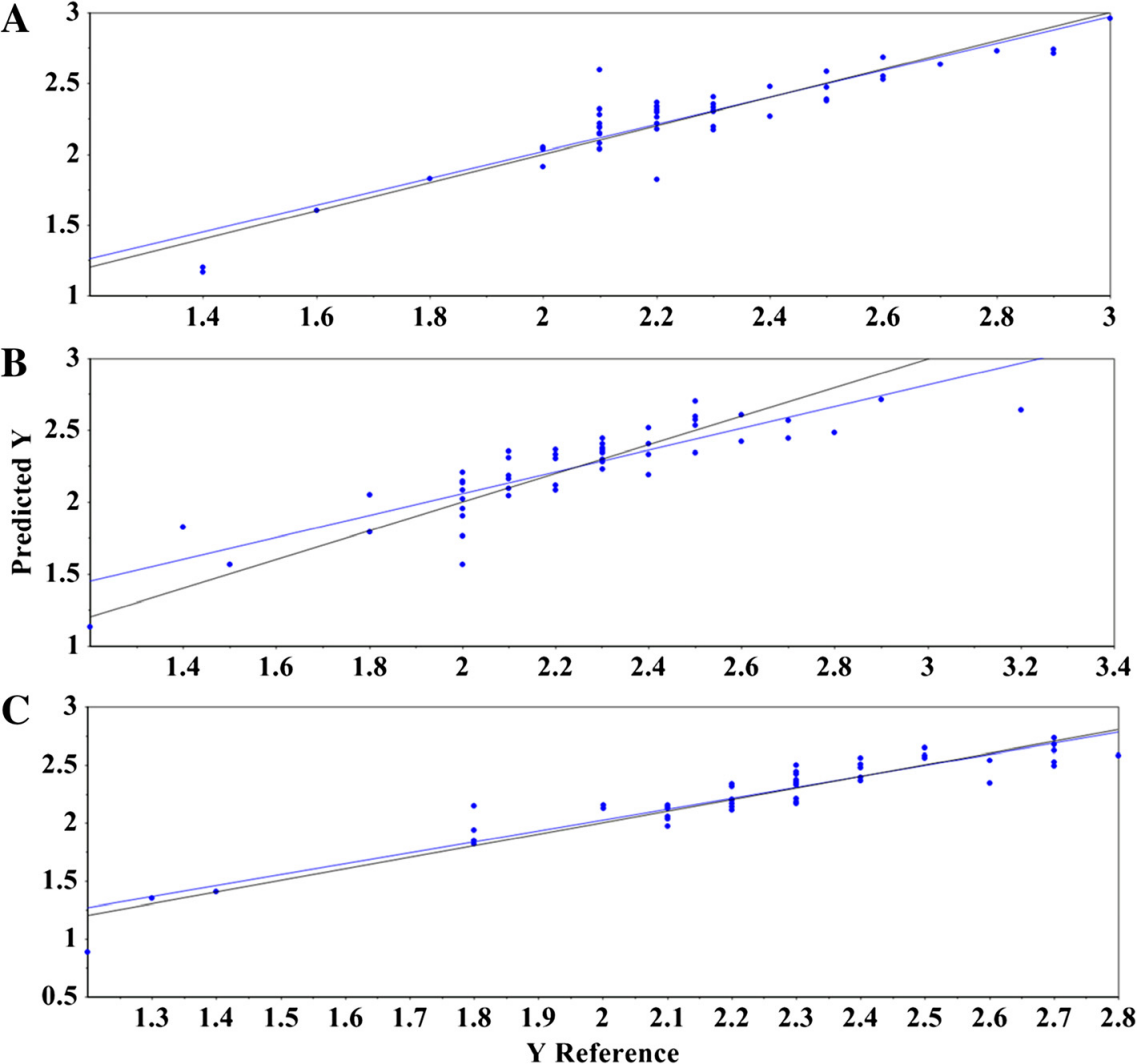
**Reference versus predicted plot for validation set using mid-infrared, near-infrared, and Raman spectral data. (A)** Plot of the predicted lignin S/G ratio using a model built from second derivative + MSC-transformed mid-infrared spectra and the reference pyMBMS data. The black line indicates the target line of optimal fit and the blue line represents the experimental fit of the data to the model. **(B)** Plot of the predicted lignin S/G ratio using a model built from second derivative + MSC-transformed near-infrared spectra and the reference pyMBMS data. The black line indicates the target line of optimal fit and the blue line represents the experimental fit of the data to the model. **(C)** Plot of the predicted lignin S/G ratio using a model built from first derivative + EMSC-transformed Raman spectra and the reference pyMBMS data. The black line indicates the target line of optimal fit and the blue line represents the experimental fit of the data to the model. The x-axis shows the pyMBMS measured lignin S/G ratio, and the y-axis reveals the predicted lignin S/G ratios. S/G = syringyl-to-guaiacyl ratio, MSC = multiplicative scatter correction, pyMBMS = pyrolysis molecular beam mass spectrometry, EMSC = extended multiplicative scatter correction.

Predictive models comprised of MIR spectra resulted in a RMSEC average of 0.13 regardless of the spectral transformations selected, and calibration *R*^*2*^ values between 0.82 ± 0.01 and 0.85 ± 0.02 (Table 2, and Additional file 1: Table S3). The models used either three or four factors to successfully explain the maximum amount of variance, without over-fitting the data. Of the 195 calibration samples, only two were characterized as outliers after thoroughly evaluating the leverage, Hotelling T^2^, residual variance, and X-Y distribution statistical plots, although this characterization was dependent on the randomized calibration and validation matrices. Thus, one of the two samples could occasionally be left in a data matrix without detrimental effects on the model. The RMSEP averages, using MIR spectroscopy were 0.13 to 0.15, with r values between 0.87 ± 0.02 and 0.91 ± 0.01 (*R*^*2*^ = 0.76 ± 0.03 to 0.83 ± 0.01, Table 3, Additional file 1: Table S3). Figure 4A demonstrates the suitability of using MIR spectroscopy to forecast lignin S/G ratios. The plot represents the best MIR model using second derivative + MSC-transformed spectral data with the pyMBMS results. The experimental trend line (blue) closely resembles the target line (black), indicating high correlation.

NIR spectroscopy, although used quite frequently in developing successful multivariate models, exhibited the highest RMSEC (0.16 to 0.17) and RMSEP (0.18 to 0.21), and lowest calibration *R*^*2*^ (0.72 ± 0.02 to 0.74 ± 0.01) and validation r values (0.79 ± 0.01 to 0.82 ± 0.04, *R*^*2*^ = 0.62 ± 0.01 to 0.67 ± 0.07) (Tables 2 and 3, Additional file 1: Table S3). The NIR spectral data required four to six factors to explain the variance and had between one and five samples characterized as an outlier, depending on the calibration and validation matrices. Samples lacking in predictive accuracy, whether in full cross-validation or randomized validation models, were left in the model unless distinguished as outliers using the aforementioned plots. Figure 4B shows the predicted versus reference plot using second derivative + MSC-transformed spectral data. The experimental trend line (blue) shows significant deviation from the target line (black).

The Raman spectral data produced PLS models with a RMSEC average of 0.13, regardless of the spectral transformations selected (Table 2, Additional file 1: Table S3). The calibration *R*^*2*^ values ranged from 0.83 ± 0.01 to 0.845 ± 0.003, four to six factors were required to explain the variance, and between two and five samples were shown to be outliers, dependent on the respective calibration and validation matrices (Table 2, Additional file 1: Table S3). The RMSEP averages ranged from 0.13 to 0.16, with r values between 0.86 ± 0.02 and 0.91 ± 0.02 (*R*^*2*^ = 0.74 ± 0.04 to 0.83 ± 0.04, Table 3, Additional file 1: Table S3). Figure 4C illustrates the competency of Raman spectroscopy to accurately predict lignin S/G ratios. In this specific model, first derivative + EMSC-transformed spectral data was coupled with the corresponding pyMBMS results. As with the MIR model (Figure 4A), the experimental trend line (blue) shows nearly superimposable correlation with the target line (black).

One of the preliminary steps in constructing a multivariate model is the mean-centering of the spectral data. An average spectrum is determined and subtracted from each individual spectrum, leaving the residual variance from the mean. It is this variance that multivariate methods splice with reference results. Figures 1, 2, and 3 show representative MIR, NIR, and Raman spectral data. The spectra have been truncated to show the regions used in the construction of the models, but have not been otherwise offset or modified. The raw Raman spectra contain substantial differences in measured vibrational modes as well as spectral intensities (Figure 3A). This is exemplified by considering the C-C stretching vibration of the polysaccharides near 1100 cm^−1^, the lignin C-C-H and –HC = CH deformation at 973 cm^−1^, and the C-O stretch, aryl symmetric bend, and C-H out of plane bend at 808 cm^−1^[35,36]. The plot of raw MIR spectra reveals greater similarities between the spectra, although there remain some spectral differences, such as the aromatic skeletal vibration at 1595 cm^−1^ (Figure 1A). Spectral differences in both the Raman and MIR spectra are further elucidated when the second derivative transformation has been applied (Figures 1B and 3B). The NIR spectra display considerable resemblance, and the lack of distinguished spectral features makes it challenging to visually differentiate between the samples (Figure 2). The second derivative NIR spectra (Figure 2B) reveal relatively few chemical signatures, especially when juxtaposed to both Raman and MIR spectral data. The accuracy of the MIR and Raman predictive models is likely correlated with the higher abundance of both spectral detail and variation.

The analysis of regression coefficient plots enables the determination of which spectral regions are deemed integral to model construction. Tables 4, 5, and 6 list the spectral regions identified by the regression coefficients plots for MIR, Raman, and NIR models, respectively, while Additional file 1: Figures S3, S4, and S5 illustrate examples of these plots graphically. The regression coefficient wavenumber regions listed encompass the range of vibrational modes used in calculating the models, but does not signify that the complete range was utilized. Tables 4, 5, and 6 demonstrate that the models successfully extracted key lignin and lignin model compound vibrational modes. Although there is overlap between some of the spectral assignments, there is general agreement between the sources as to peak location, and their classification as bonds representative of lignin and lignin monomers. It should be noted that different instrumental configurations can lead to variation in vibrational mode peak locations.

**Table 4:** MIR vibrational mode regions identified from regression coefficient plots and spectral assignments corresponding to lignin and/or lignin monomers

**Vibrational mode from regression coefficients plot**	**S/G/H vibrational mode and spectral assignment**
788-790	784 (G) [37]
808-836	813 (G) [37]
	827 (S) [38]
854-883	863, 878 (G) [37]
912-917	914 (G) [37]
1137-1168	1142 (G) [39]
	1151 (G) [40]
1205-1263	1215 (Lignin) [40]
	1226 (G), 1252 [39]
1270-1299	1270 (G) [39]
	1269 (G) [40]
1319-1425	1425 (S) [38]
	1330, 1425 (S), 1379, 1428 (Lignin) [39]
	1327 (G), 1425, 1427 [40]
1442-1502	1500 (S) [38]
	1465 (Lignin) [39]
	1462, 1463 [40]
1508-1521	1506-1513 [39]
	1513, 1514 [40]
1585-1606	1589 (S) [38]
	1596–1600 (Lignin) [39]
	1594, 1603 [40]
1610-1612	1610 [41]
1698-1714	1704 [40]
1745-1756	1733-1753 [42]

G = guaiacyl, S = syringyl.

**Table 5:** Raman vibrational modes identified from regression coefficient plots and spectral assignments corresponding to lignin and/or lignin monomers

**Vibrational mode from regression coefficients plot**	**S/G/H vibrational mode and spectral assignment(s)**
351-376	369 (S), 357, 370 (G) [35]
378-401	370-399 (S) [20]
474-623	529, 564, 582 (S), 541, 559, 590 (G) [35]
665-725	711 (S) [43]
	712 (G), 701 (H) [35]
736-756	741 (S) [43]
	741 (H) [35]
748-765	761 (G) [43]
765-796	781-820 (S) [20]
	784 (G) [43]
	793 (G) [35]
800-835	819-864 (H) [20]
	810 (S) [43]
	799 (S), 823 (H) [35]
875-939	920 (G) [43]
	907 (S), 921 (G) [35]
991-1051	1024 (G) [43]
	1043 (S), 1036 (G) [35]
1091-1131	1108 (S), 1124 (G), 1094 (H) [43]
	1116 (S), 1122 (G), 1105 (H) [35]
1135-1195	1154 (S), 1158 (G), 1168 (H), 1170 (Lignin) [28]
	1138–1160 (S), 1162–1188 (G), 1163–1179 (H) [20]
	1148 (S), 1186 (G), 1164 (H) [43]
	1152, 1187 (S), 1155, 1186 (G), 1173, 1199 (H) [35]
1205-1242	1200 (H) [28]
	1213–1218 (H) [20]
	1228 (S), 1215 (H) [43]
	1214, 1241 (S), 1208, 1241 (G), 1216 (H) [35]
1261-1346	1337 (S), 1263 (H), 1270 (Lignin) [28]
	1262–1275 (G), 1318–1332, 1331–1338 (S), 1286–1299 (H) [20]
	1331 (S), 1270–1285 (G), 1338 H [43]
	1331 (S), 1272, 1288 (G), 1298, 1331 (H) [35]
1434-1448	1454-1460 (S), 1452–1465 (G), 1452–1459 (H) [20]
	1452 (S), 1455 (G), 1455 (H) [35]
1587-1606	1594 (S), 1589, 1604 (G), 1588, 1606 (H), 1591, 1604 (Lignin) [28]
	1588 (S) [43]
	1609 (S), 1609 (G), 1599 (H) [35]
1623-1629	1634 (S), 1633 (G), 1632 (H), 1634 (Lignin) [28]
1653-1672	coniferyl (G) and sinapyl (G) alcohol [44]

G = guaiacyl, S = syringyl, H = p-coumaryl.

**Table 6:** NIR vibrational modes identified from regression coefficient plots and spectral assignments corresponding to lignin and/or lignin monomers

**Vibrational mode from regression coefficients plot**	**Lignin vibrational mode**
4400-4586	4411 [45]
	4546 [46]
	4686 [47]
5581-5600	5583 [45]
5959-6009	5963, 5978 [45]
	5974, 5978, 5980 [46]
7081-7197	7092 [48]
8459-8674	8547 [46]
8720-8801	8749 [49]

The most intense vibrational modes of cellulose occur at 1091 and 1117 cm^−1^[36]. While these, and less intense peaks indicative of cellulose, are encompassed in the spectral regions identified by the MIR and Raman regression coefficients plots, further analysis revealed that the known cellulose or polysaccharide vibrational modes (such as 1091 cm^−1^), were negatively correlated (for example, Additional file 1: Figure S4) or were not identified as important to the model construction (896, 1074, and 1268 cm^−1^). A positive correlation was determined for peaks due to cellulose and lignin vibrational modes (for example, 1117 and 1338 cm^−1^). The NIR regression coefficients plots contained various regions that were not attributable to lignin and lignin derived molecules besides those itemized in Table 6. Given the limited spectral diversity measured using NIR, since only overtone and combination bands are evaluated, interpreting regression coefficients plots becomes more ambiguous regarding what is being predicted.

To summarize, the RMSEP values measured in MIR and Raman models (0.13 to 0.16, approximately two to three times higher than the SEL) did not statistically differ, indicative that either spectroscopic technique could be used to obtain similar results. The NIR models resulted in a RMSEP of 0.18 to 0.21 (three to four times higher than the SEL. This increase in error may not warrant migration from using NIR spectroscopy as an analytical tool for developing predictive multivariate models however, especially when factors such as instrument expense and ease of use are considered. Ultimately, identifying other useful applications of the MIR and Raman instrumentation (for example, structural analysis) will aid in selecting which analytical tool(s) will be appropriate.

The models produced in this study contain 17 different plant species across three genera. Rather than assemble three separate models for each genus, a more global approach was undertaken to construct one robust model capable of predicting both heartwood and sapwood samples from *Acacia*, *Corymbia*, and *Eucalyptus*. Additionally, the metrics listed in Tables 2 and 3 are the average results obtained from using three randomized calibration and validation matrices for each spectral transformation (Additional file 1: Table S3). This tactic permitted the development of completely independent predictive models, whereas the use of one set of calibration and validation matrices for all spectroscopic data sets may have introduced a level of undesirable bias into the models.

## Conclusions

Eucalypts, including *Corymbia* and *Eucalyptus*, and *Acacias* present an attractive option for biofuel and biochemical production. Given the pool of over 900 diverse species of eucalypts, it is essential to isolate biomass species possessing traits found to play important roles in diminishing biomass recalcitrance. The standard methods of analysis are time-consuming, potentially toxic, and can destroy the sample. The use of multivariate modeling can significantly reduce experiment and analysis time and expense. This research has illustrated the validity of vibrational spectroscopy to provide non-destructive, accurate, global, predictive models encompassing an assorted array of feedstocks for the determination of lignin S/G ratios. Models constructed using MIR and Raman spectral data resulted in more accurate predictions compared to those produced from NIR spectra (RMSEP of 0.13 to 0.16 for MIR and Raman versus 0.18 to 0.21 for NIR). Current investigations are also underway to apply vibrational spectroscopy and PLS modeling for the prediction of cell-wall composition, including cellulose, xylan, and lignin content, and projected glucose and xylose release.

## Methods

### Wood samples

The 245 wood samples were obtained from a range of subtropical and temperate tree species either currently used for pulp or timber plantations or were prospective species for biomass and/or oil production [34]. The trees studied were as follows: *Corymbia citriodora* subspecies *citriodora, Corymbia torelliana*, *Corymbia citriodora* subspecies *variegata*, several families of *Corymbia* hybrids [50], *Eucalyptus argophloia*, *Eucalyptus cloeziana*, *Eucalyptus crebra*, E*ucalyptus dunnii*, *Eucalyptus globulus* subsp. *maidenii*, *Eucalyptus grandis*, *Eucalyptus kochii*, *Eucalyptus longirostrata*, *Eucalyptus loxophleba*, *Eucalyptus moluccana*, *Eucalyptus polybractea*, *Acacia microbotrya*, and *Acacia saligna*. Trees were selected from trial sites in New South Wales (1), Western Australia (3) or Queensland (4) (Additional file 1: Table S1). Trees from New South Wales and Queensland sites were from Queensland Department of Agriculture, Fisheries and Forestry trials. They were sampled around 10 years of age except for the Narayan site (Additional file 1: Table S1) which was sampled at age 19 years. The trees chosen for this study stratified the size classes of the species in the trials and the samples were collected from 10 to 15 trees per species per site to ensure the full species diversity for wood property traits was captured. Approximately 2 to 3 g of drill swarf was obtained by drilling a 4 cm hole in the main stem at breast height (between 1.2 and 1.4 m height) in each tree using a 16 mm spade bit on a battery powered drill, after removing the outer-bark. Swarf was collected into paper bags and allowed to dry to ambient moisture levels in an air conditioned room for at least 48 hours prior to transfer into plastic vials (10 ml Falcon tubes) for storage and shipment. Similar sampling techniques were used for the material from Western Australia. All samples were ground for five minutes using the Joint BioEnergy Institute (Sandia National Laboratories) Biomass Preparation System robot created at Labman Automation Ltd. (North Yorkshire, UK). The samples were oven-dried at 105°C for approximately 30 minutes to decrease moisture content before the spectra were collected.

### Fourier-transform infrared spectroscopy

MIR spectra of raw biomass were collected with a Bruker Vertex 70 (Billerica, Massachusetts, United States) spectrometer, equipped with a HTS-XT 96-well microplate reader (Bruker, Billerica, Massachusetts, United States). Each spectrum was acquired in absorbance mode using 96 scans and a spectral resolution of 4 cm^−1^. Three representative spectra were averaged for each sample using the Bruker OPUS software package (Billerica, Massachusetts, United States). All other spectral processing was performed using the Unscrambler X (Camo Inc., Oslo, Norway). The MIR spectra (Figure 1) were collected using 96 scans, as more scans did not lead to a visually significant reduction of noisy spectral regions (data not shown). There is a tradeoff between the number of scans and the amount of time spent acquiring the spectra. A 96-well plate could be measured in approximately 48 minutes (30 seconds per sample) using 96 scans per sample.

### Near-infrared spectroscopy

NIR spectra of raw biomass were collected using a Bruker FT-NIR Multi-Purpose Analyzer (Billerica, Massachusetts, United States) in diffuse reflectance mode, also equipped with the HTS-XT 96-well microplate reader. Each spectrum was acquired using 256 scans and a spectral resolution of 8 cm^−1^. Three representative spectra were averaged for each sample using the Bruker OPUS software package. All other spectral processing was performed using the Unscrambler X. The NIR spectra (Figure 2) were collected using 256 scans, since the signal was intrinsically much weaker than MIR spectral intensities due to the excitation of combination and overtone, rather than fundamental vibrational modes. Additionally, the use of the 96-well plate resulted in lower spectral intensities compared to measuring the samples individually in glass vials. Using 256 scans per sample, a accept plate could be measured with NIR spectroscopy in approximately one hour.

### Fourier-transform Raman spectroscopy

Raman spectra (Figure 3) of raw biomass were collected with a Bruker MultiRAM Stand Alone Spectrometer (Billerica, Massachusetts, United States) equipped with a HTS-mapping stage for measuring samples in glass-bottomed 96-well plates. The 1064 nm tunable Nd:YAG laser was programmed to 350 mW to maximize the Raman scatter. Each spectrum was acquired using 32 and 96 scans, with a spectral resolution of 4 cm^−1^. Three representative spectra were averaged for each sample using the Bruker OPUS software package. All other spectral processing was performed using the Unscrambler X. The total analysis time was kept at approximately one hour by employing 32 spectral scans. An additional data set using 96 scans was collected to provide a more direct comparison between MIR and Raman spectra. A 96-well plate could be measured in about five hours using 96 scans (about three minutes per sample).

### Reference data set

The spectral pre-processing for PCA included baseline correction and either seven- (NIR) or nine-point (MIR) Savitzky-Golay smoothing. The resultant PCA allowed the rough classification of ‘unique’ plant samples, as determined by using the scores, Hotelling T^2^, leverage, and residual variance plots. The reference data set was composed of the ‘unique’ plants established from the PCA metrics, and a random selection of feedstocks representative of the majority of the samples. The spectral regions used for PCA analysis were 8794 to 3999 cm^−1^ for NIR and 5303 to 599 cm^−1^ for MIR.

### Pyrolysis/molecular beam mass spectrometry

A custom built pyrolysis/molecular beam mass spectrometer (pyMBMS), at the National Renewable Energy Lab (Golden, Colorado, USA), was used as the reference technique for quantifying S and G lignin ratios. The instrumental details have been previously reported [4]. A full description is available in Additional file 1. The peaks representative of lignin were those with mass-to-charge ratio (m/z) = 120, 124, 137, 138, 150, 152, 154, 164, 167, 178, 180, 181, 182, 194, and 210. S/G ratios were calculated by dividing the sum of the syringyl peaks at 154, 167, 168, 182, 194, 208, and 210 by the sum of guaiacyl peaks at 124, 137, 138, 150, 164, and 178. It is worth noting that a few lignin peaks had correlations with both S and G precursors, and subsequently were left out of the S and G calculations.

### Multivariate analysis

The spectral data (MIR, NIR, and Raman, Figures 1, 2, and 3) was imported from OPUS into the Unscrambler X. A variety of spectral processing techniques were employed to see which methods appropriately corrected for additive and multiplicative effects, such as baseline offsets and particle size variations. The spectral transformations included: first or second Savitzky-Golay derivatives, standard normal variate (SNV), multiplicative scatter correction (MSC), and extended MSC (EMSC), as well as combinations of a derivative and SNV, MSC, or EMSC. Since the derivative transformations increase spectral noise, iterations of varying degrees of spectral smoothing were tested to see which reduced noise without sacrificing signal.

Reference sets of 195 randomly selected samples were used for PLS model construction. Each model was built using transformed spectral data that had been mean-centered. The regression coefficients plot was used to identify which spectral variables were paramount in constructing the model, and recalculation of each model by using the marked important variables removed featureless spectral regions, thereby reducing spectral noise in the model. A full cross-validation was used to evaluate how the spectral transformations affected model accuracy. Validation sets of 50 randomly selected reference samples (separate from the 195 calibration samples) were used to evaluate the accuracy and robustness of the model’s predictive capacity. The randomization was achieved in Microsoft Excel, using the random number generator function. This was performed three times for each method of spectroscopic processing. For example, three randomly generated calibration and validation matrices were combined with the Raman spectral data that had been transformed using a first derivative and EMSC. As shown in Additional file 1: Table S3, the models created using this procedure were not statistically different.

## Abbreviations

EMSC: Extended Multiplicative Scatter Correction; GC/MS: Gas Chromatography/Mass Spectrometry; MIR: Mid-infrared Spectroscopy; MSC: Multiplicative Scatter Correction; NIR: Near-infrared Spectroscopy; PCA: Principal Component Analysis; PLS: Partial Least Squares Regression; pyMBMS: Pyrolysis Molecular Beam Mass Spectrometry; r: Coefficient of Correlation for Validation Set; *R*^2^Cal: Coefficient of Determination for Calibration Set; *R*^2^CV: Coefficient of Determination for Full Cross-Validation; *R*^2^Val: Coefficient of Determination for Validation Set; RMSEC: Root Mean Standard Error of Calibration; RMSECV: Root Mean Standard Error of Cross-Validation; RMSEP: Root Mean Standard Error of Prediction; SEL: Standard Error of the Laboratory; SEP: Standard Error of Prediction; SNV: Standard Normal Variate.

## Competing interests

The authors declare that they have no competing interests.

## Authors’ contributions

JSL designed all spectroscopic methodology, collected the spectral data, created the multivariate analysis models, and wrote the manuscript. SS and BAS provided guidance throughout all experimentation and critically revised the manuscript. MD provided assistance with the multivariate analysis. DL and MS selected and collected the majority of the wood samples for analysis and provided information regarding the environmental and growing conditions. RH conceived the project, provided the funding, and critically revised the manuscript. All authors read and approved the final manuscript.

## Supplementary Material

Additional file 1: Table S1 Environmental characteristics for the plant growing sites. **Table S2.** Lignin S/G ratios as determined by pyrolysis molecular beam mass spectrometry. **Table S3.** Individual model parameters. **Figure S1.** MIR (top) and NIR (bottom) scores plots used to determine ‘unique’ samples. **Figure S2.** Example of residual variance or Scree plot used in determining the appropriate number of factors. **Figure S3.** Example of MIR regression coefficient plots used to determine which spectral regions used to construct the models. **Figure S4.** Example of Raman regression coefficient plots used to determine which spectral regions used to construct the models. **Figure S5.** Example of NIR regression coefficient plots used to determine which spectral regions used to construct the models. Click here for file
