# Supplementary material for: High-throughput prediction of eucalypt lignin syringyl/guaiacyl content using multivariate analysis: a comparison between mid-infrared, near-infrared, and Raman spectroscopies for model development
Source: Biotechnol Biofuels. 2014 Jun 17;7:93. doi: 10.1186/1754-6834-7-93 (PMC4064109; doi:10.1186/1754-6834-7-93)
Supplement: Additional file 1: Table S1 — Environmental characteristics for the plant growing sites. Table S2. Lignin S/G ratios as determined by pyrolysis molecular beam mass spectrometry. Table S3. Individual model parameters. Figure S1. MIR (top) and NIR (bottom) scores plots used to determine ‘unique’ samples. Figure S2. Example of residual variance or Scree plot used in determining the appropriate number of factors. Figure S3. Example of MIR regression coefficient plots used to determine which spectral regions used to construct the models. Figure S4. Example of Raman regression coefficient plots used to determine which spectral regions used to construct the models. Figure S5. Example of NIR regression coefficient plots used to determine which spectral regions used to construct the models. [file 1754-6834-7-93-S1.docx]

**High-throughput prediction of eucalypt lignin syringyl/guaiacyl content using multivariate analysis: a comparison between mid-infrared, near-infrared, and Raman spectroscopies for model development**

**Additional Information**

**Jason S. Lupoi^1,2*^, Seema Singh^2,3^, Mark Davis^4,5^, David J. Lee^6^. Merv Shepherd^7^, Blake A. Simmons^1,2,3^ , and Robert J. Henry^1^**

^1^Queensland Alliance for Agriculture and Food Innovation, The University of Queensland, St. Lucia, Queensland, 4072, Australia

^2^Joint BioEnergy Institute, Lawrence Berkeley National Laboratory, 5885 Hollis Street, Emeryville, California, 94608, United States of America

^3^Biological and Materials Science Center, Sandia National Laboratories, 7011 East Avenue, Livermore, California, 94551, United States of America

^4^BioEnergy Science Center, Oak Ridge National Laboratory, 1 Bethel Valley Rd, Oak Ridge, Tennessee 37831, United States of America

^5^National Bioenergy Center, National Renewable Energy Laboratory, 15013 Denver West Parkway, Golden, Colorado 80401, United States of America

^6^Forest Industries Research Centre, University of the Sunshine Coast and Queensland Department of Agriculture, Fisheries and Forestry, Locked Bag 4, Maroochydore DC, Queensland, 4558, Australia

^7^Southern Cross Plant Science, Southern Cross University, Military Road, Lismore NSW, Australia

Contact Information: (email) jslupoi@lbl.gov*

seesing@sandia.gov

[Mark.Davis@nrel.gov](mailto:Mark.Davis@nrel.gov)

dlee@usc.edu.au

mervyn.shepherd@scu.edu.au

[basimmons@lbl.gov](mailto:basimmons@lbl.gov)

[robert.henry@uq.edu.au](mailto:robert.henry@uq.edu.au)

**Pyrolysis molecular beam mass spectrometry**

The instrument uses an Extrel Model TQMS C50 mass spectrometer (Extral Core Mass Spectrometers, Pittsburgh, PA) coupled with a Frontier model PY-2020 iD autosampler (Frontier Laboratories Ltd., Fukushima, Japan). Approximately four milligrams of ground biomass was placed into each compartment of a 48-well tray. All samples were measured twice. The sample was introduced to the mass spectrometer via helium gas using a 2.0 L/min flow rate. The autosampler furnace was set to a pyrolysis temperature of 500°C. The transfer line interfacing the pyrolyzer to the spectrometer was set to 350°C. The total pyrolysis time was 2 minutes.

Spectra generated using pyMBMS were background corrected using the Merlin Automation Data System, version 2.0 (Extrel). The data was transferred to Microsoft Excel for formatting, and then imported into the Unscrambler (version 9.7) where the spectra were normalized to the total ion current to account for variation in the sample masses pyrolyzed.

**Additional Table 1. Environmental characteristics for the plant growing sites**

| **Site** | **State** | **Longitude/Latitude** | **Average Annual Rainfall (mm)** | **Soil** | **Slope** |
| --- | --- | --- | --- | --- | --- |
| Amamoor | Queensland | 152.53° -26.36° | 1090 | Black Dermasol (close to yellow podzolic) | 0° |
| Cuballing Rd | W. Australia | 117.18°  -32.92° | N/A | Loamy gravel | N/A |
| Dwarda | W. Australia | 116.68°  -32.77° | N/A | Pale deep sand | N/A |
| Hills | New South Wales | 153.05º  -28.61º | 1082 | Prairie soils (Northcote classification Gn3.93) | 10.5º WSW |
| McKenzies | Queensland | 148.96º  -21.00º | 1700 | Brown Dermasol | 0-5º SE |
| Mary Smokes | Queensland | 152.68º  -26.93º | 1020 | Siliceous sand to grey podzolic | 8º ESE |
| Narayan | Queensland | 150.87º  -25.70º | 716 | Grey chromosol | 0-5º SW |
| Rhodes Farm | W. Australia | 117.25°  -32.88° | 431 | Loamy gravel | 1.22 |
| Thompsons Farm | W. Australia | 117.15°  -32.97° | 460 | Moderately deep sandy gravel | 0.67 |
| Verve Farm | W. Australia | 117.19°  -32.95° | 449 | Loamy gravel | 1.30 |

**Additional Table 2. Lignin S/G ratios as determined by Pyrolysis Molecular Beam Mass Spectrometry**

| **Plant Species** | **Lignin S/G Ratio Average** | **Standard**  **Deviation** | **S/G Range** | **# of Samples** |
| --- | --- | --- | --- | --- |
| *Acacia microbotrya* | 1.3 | 0.1 | 1.2-1.5 | 5 |
| *A. saligna* | 1.7 | 0.2 | 1.4-1.9 | 4 |
| *Corymbia citriodora* subsp. *citriodora* | 2.4 | 0.2 | 2.1-2.8 | 17 |
| *C. citriodora variegata* | 2.3 | 0.1 | 2.0-2.5 | 17 |
| *Corymbia* hybrids  (sensu Lee 2007) | 2.3 | 0.2 | 1.6-2.8 | 47 |
| *C. torelliana* | 2.1 | 0.1 | 1.8-2.4 | 56 |
| *C. citriodora* subsp.*variegata* | 2.6 | 0.2 | 2.0-3.2 | 22 |
| *Eucalyptus argophloia* | 2.1 | 0.1 | 1.9-2.2 | 5 |
| *E. cladocalyx* | 2.5 | 0.2 | 2.3-2.6 | 3 |
| *E. cloeziana* | 1.9 | 0.2 | 1.7-2.3 | 7 |
| *E. crebra* | 1.6 | 0.4 | 1.4-2.1 | 4 |
| *E. dunnii* | 2.5 | 0.3 | 2.2-2.8 | 4 |
| *E. globulus* | 2.6 | 0.2 | 2.3-3.0 | 11 |
| *E. grandis* | 2.0 | 0.2 | 1.9, 2.2 | 2 |
| *E. kochii* | 2.2 | 0.2 | 1.9-2.3 | 5 |
| *E. longirostrata* | 2.2 | 0.1 | 2.1-2.4 | 8 |
| *E. loxophleba* | 2.4 | 0.1 | 2.1-2.6 | 7 |
| *E. moluccana* | 2.2 | 0.2 | 2.0-2.5 | 5 |
| *E. occidentalis* | 2.4 | 0.2 | 2.1-2.5 | 6 |
| *E. polybractea* | 2.3 | 0.2 | 2.0-2.7 | 8 |

Lee, D. J. (2007). Achievements in forest tree improvement in Australia and New Zealand 2: Development of Corymbia species and hybrids for plantations in eastern Australia. *Australian Forestry* 70(1): 11-16.

**Additional Figure 1. MIR (top) and NIR (bottom) scores plots used to determine “unique” samples**

**
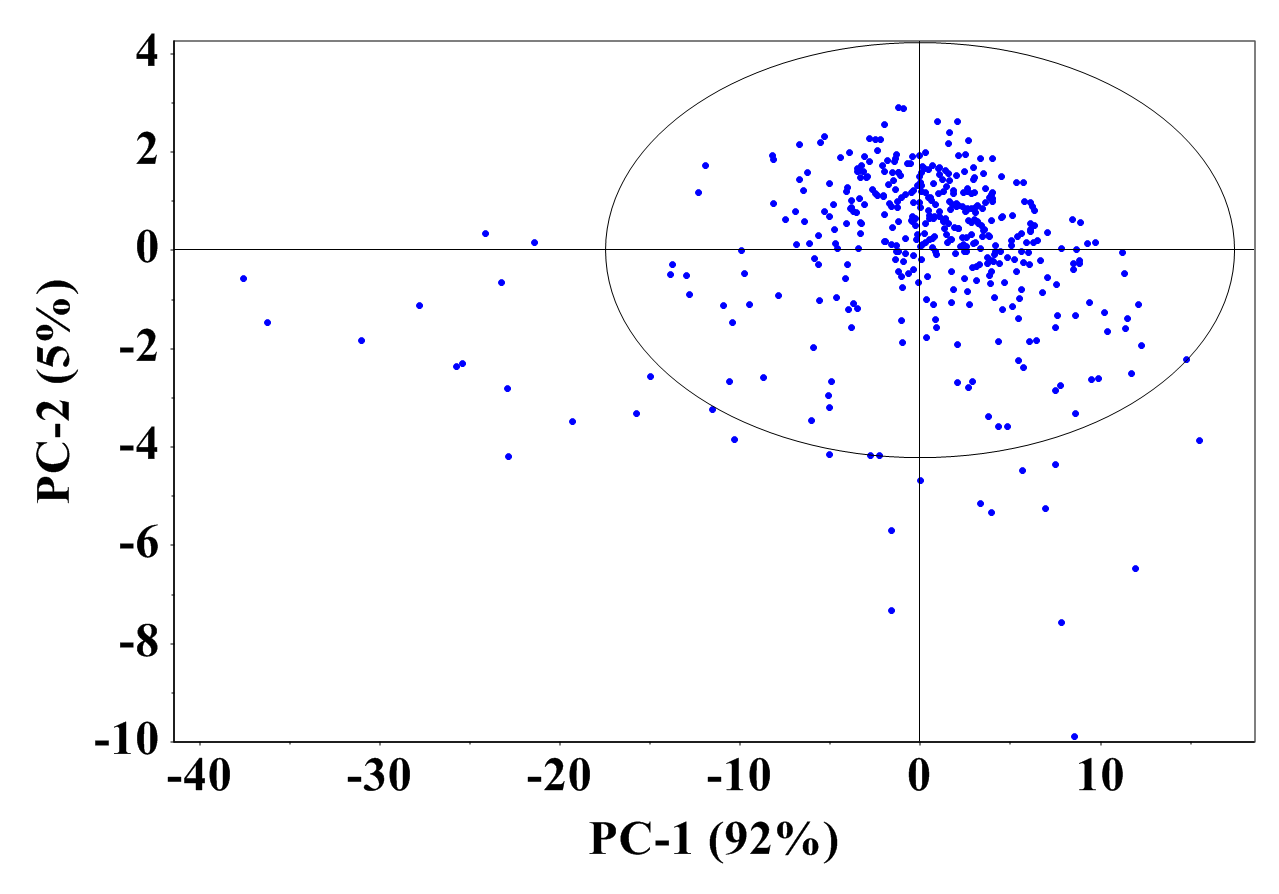

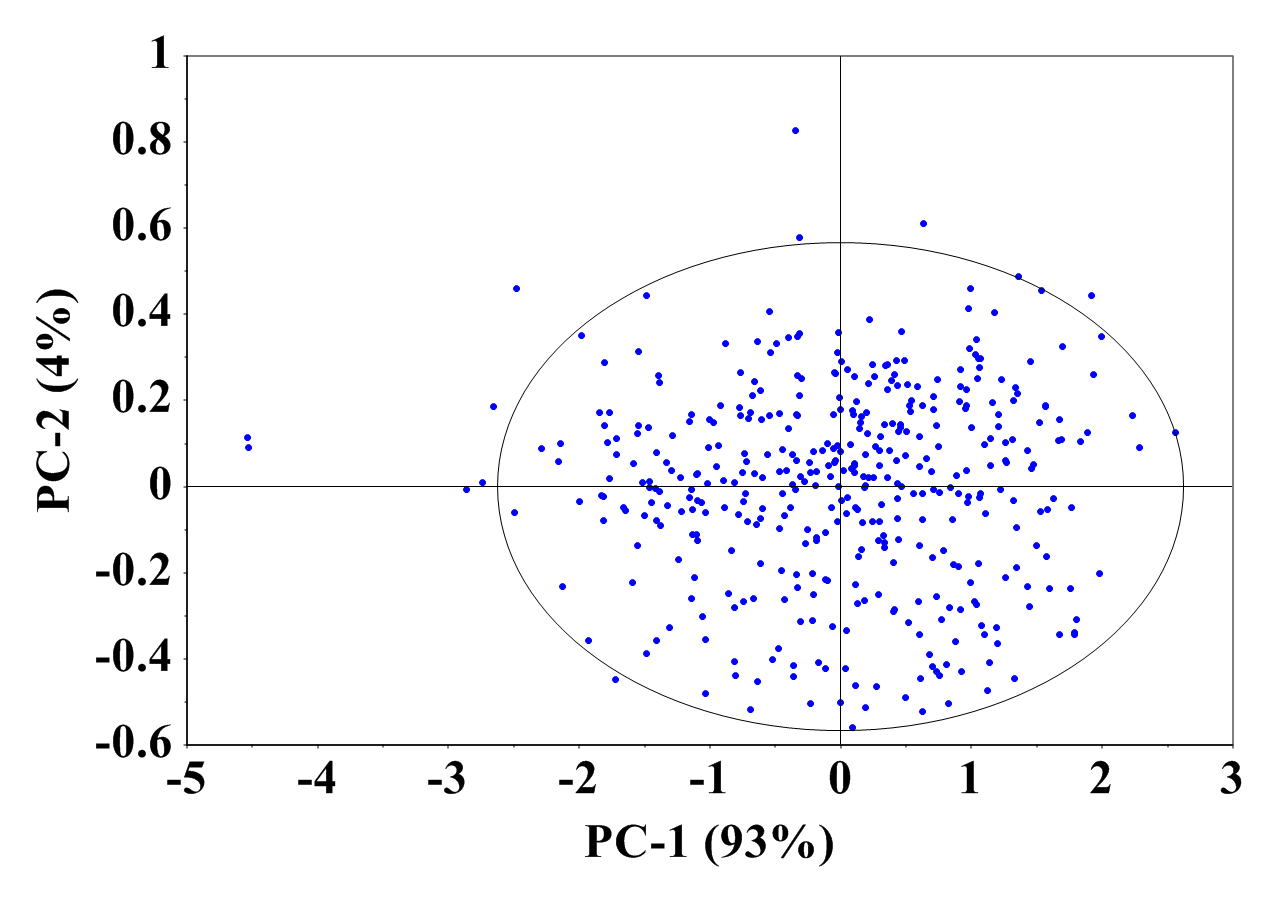
**

**Additional Figure 2. Example of residual variance or Scree plot used in determining the appropriate number of factors.**

**
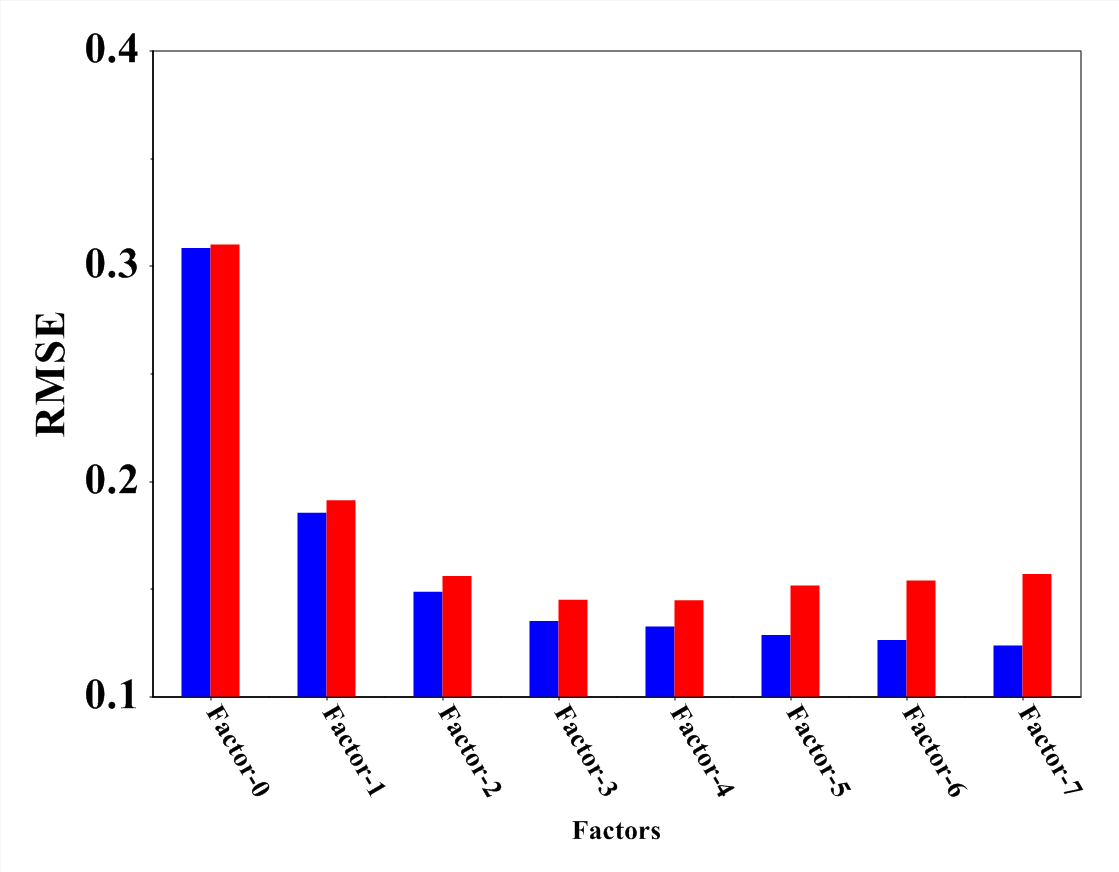
**

**Additional Figure 3**. **Example of MIR regression coefficient plots used to determine which spectral regions used to construct the models.**

**
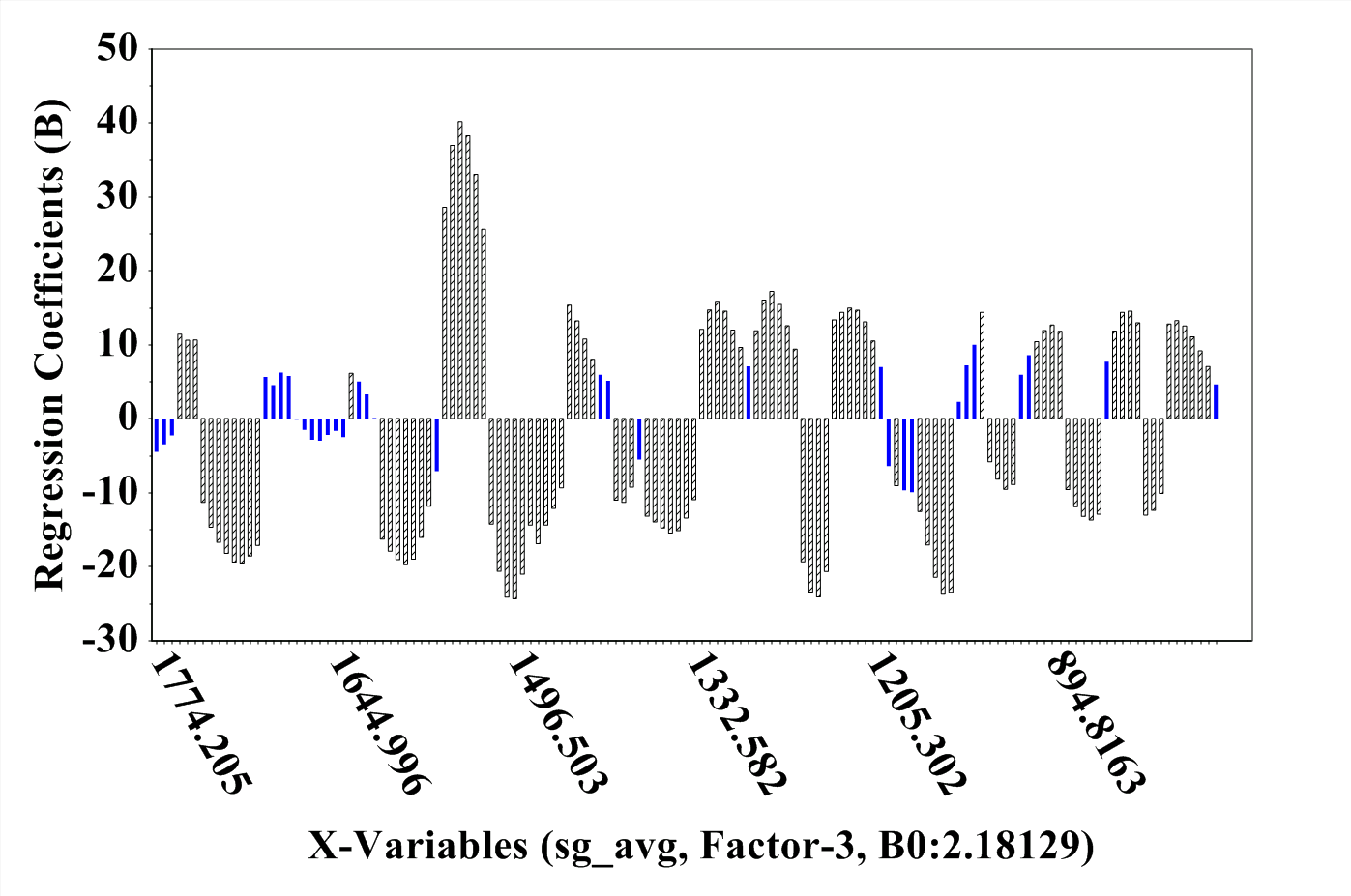
**

**Additional Figure 4**. **Example of Raman regression coefficient plots used to determine which spectral regions used to construct the models.**

**
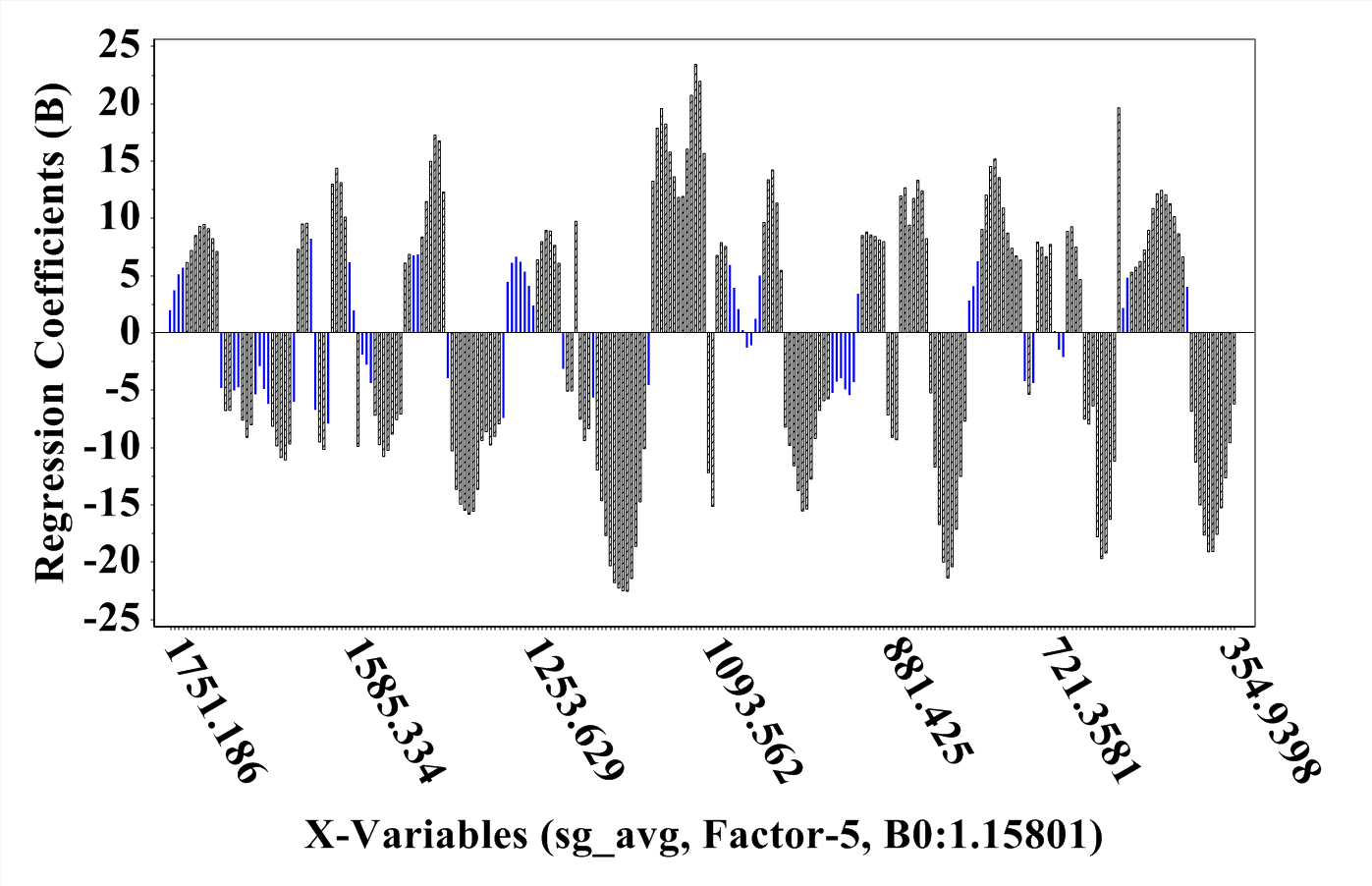
**

**Additional Figure 5**. **Example of NIR regression coefficient plots used to determine which spectral regions used to construct the models.**

**
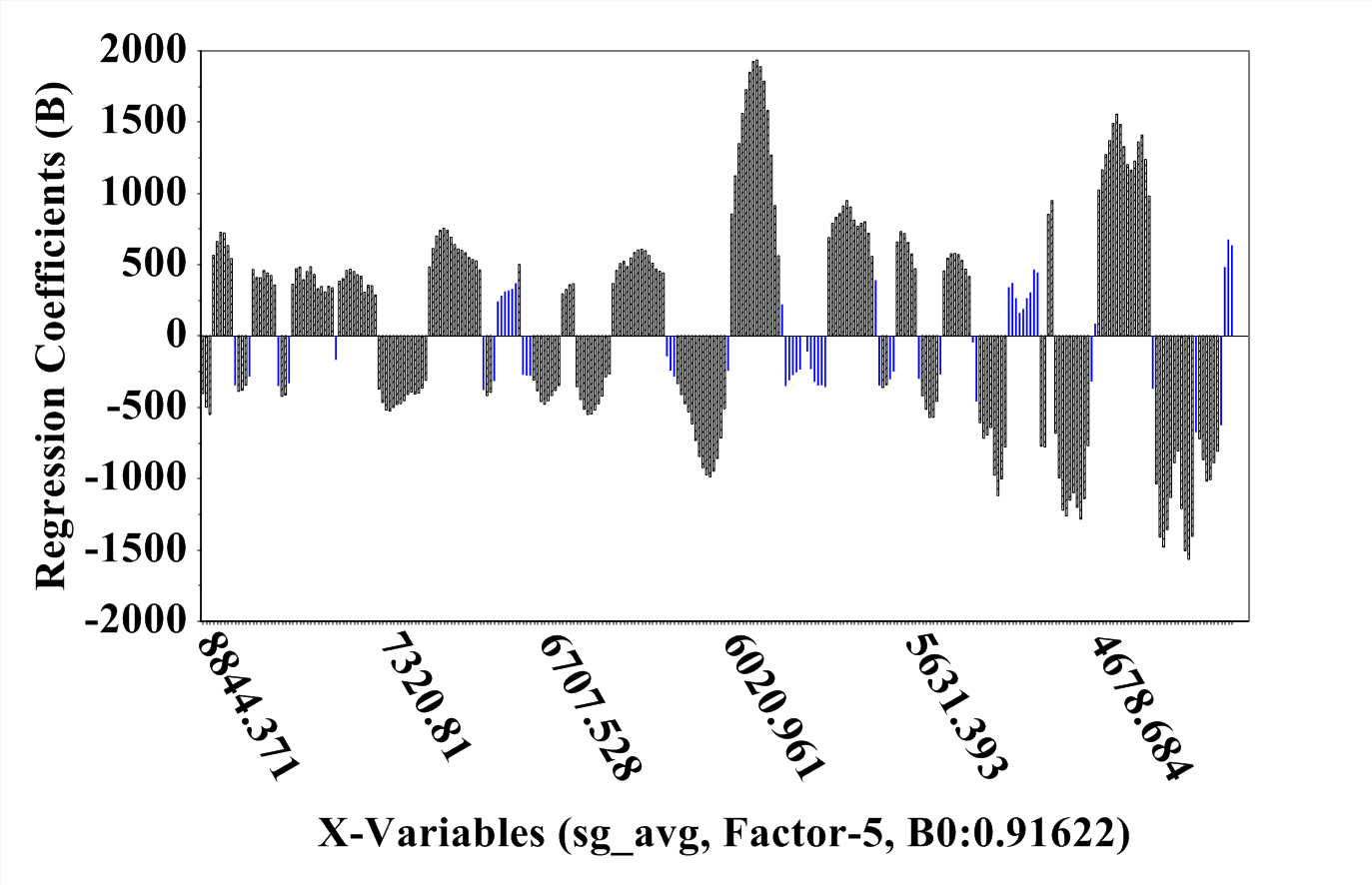
**

**Additional Table 3. Individual model parameters**

| **Method** | **SEL Cal** | **SEL Val** | **RMSEC** | **RMSECV** | **RMSEP** | **R^2^Cal** | **R^2^Val** | **Slope** | **Offset** | **Outliers** |
| --- | --- | --- | --- | --- | --- | --- | --- | --- | --- | --- |
| **Raman**  **2^nd^ deriv (19pt) +SNV;**  **32 scans** | 0.05  0.05  0.05 | 0.05  0.05  0.06 | 0.13  0.13  0.14 | 0.14  0.14  0.15 | 0.12  0.13  0.15 | 0.84  0.84  0.81 | 0.82  0.86  0.71 | 0.840  0.982  0.698 | 0.352  0.065  0.823 | 1-Val  2-Cal  2-Cal |
| **Raman**  **1^st^ deriv (7pt)+EMSC;**  **32 scans** | 0.05  0.06  0.05 | 0.06  0.04  0.06 | 0.12  0.13  0.13 | 0.13  0.14  0.14 | 0.12  0.12  0.14 | 0.85  0.84  0.84 | 0.87  0.79  0.82 | 0.946  0.899  0.688 | 0.134  0.227  0.705 | 3-Cal, 1-Val  3-Cal  1-Val |
| **Raman**  **EMSC+2^nd^ deriv (15pt);**  **96 scans** | 0.05  0.06  0.05 | 0.06  0.05  0.05 | 0.13  0.13  0.13 | 0.14  0.14  0.13 | 0.13  0.16  0.16 | 0.83  0.84  0.83 | 0.84  0.76  0.83 | 0.814  0.664  0.898 | 0.390  0.740  0.118 | 3-Cal  2-Cal  2-Cal |
| **Raman**  **2^nd^ deriv (15pt)+SNV;**  **96 scans** | 0.05  0.05  0.05 | 0.06  0.06  0.05 | 0.13  0.12  0.13 | 0.14  0.12  0.14 | 0.15  0.19  0.15 | 0.83  0.85  0.83 | 0.73  0.78  0.71 | 0.708  0.833  0.669 | 0.640  0.370  0.744 | 5-Cal  4-Cal  4-Cal |
| **MIR**  **EMSC+2^nd^ deriv (15pt)** | 0.05  0.06  0.05 | 0.06  0.04  0.05 | 0.12  0.14  0.14 | 0.13  0.15  0.15 | 0.15  0.12  0.13 | 0.87  0.83  0.82 | 0.65  0.82  0.82 | 0.839  0.770  0.819 | 0.378  0.507  0.390 | 1-Cal, 1-Val  2-Cal  2-Cal |
| **MIR**  **2^nd^deriv (17pt)+MSC** | 0.05  0.05  0.05 | 0.06  0.05  0.06 | 0.14  0.13  0.13 | 0.15  0.14  0.14 | 0.14  0.14  0.15 | 0.81  0.83  0.82 | 0.82  0.84  0.83 | 0.847  0.950  0.783 | 0.329  0.122  0.474 | 1-Cal  2-Cal, 1-Val  2-Cal, 1-Val |
| **MIR**  **2^nd^ deriv (17pt)+SNV** | 0.05  0.05  0.06 | 0.05  0.05  0.04 | 0.14  0.12  0.11 | 0.15  0.14  0.13 | 0.13  0.17  0.16 | 0.83  0.86  0.87 | 0.76  0.73  0.80 | 0.757  0.673  0.795 | 0.538  0.700  0.430 | 3-Cal, 1-Val  2-Cal, 1-Val  3-Cal |
| **NIR**  **EMSC+2^nd^ deriv(25pt)** | 0.05  0.05  0.05 | 0.05  0.06  0.05 | 0.17  0.16  0.18 | 0.18  0.18  0.19 | 0.20  0.23  0.17 | 0.73  0.73  0.72 | 0.63  0.63  0.61 | 0.637  0.668  0.645 | 0.784  0.662  0.785 | 4-Cal  5-Cal  4-Cal |
| **NIR**  **2^nd^ deriv(25pt)+**  **MSC** | 0.05  0.05  0.05 | 0.05  0.06  0.07 | 0.16  0.17  0.17 | 0.18  0.19  0.18 | 0.17  0.18  0.20 | 0.74  0.70  0.73 | 0.64  0.75  0.62 | 0.620  0.780  0.718 | 0.850  0.541  0.637 | 1-Cal, 1-Val  4-Cal  4-Cal |
| **NIR**  **2^nd^ deriv(25pt)+**  **SNV** | 0.05  0.05  0.05 | 0.05  0.06  0.06 | 0.16  0.16  0.16 | 0.17  0.17  0.16 | 0.22  0.20  0.20 | 0.76  0.73  0.73 | 0.57  0.68  0.70 | 0.550  0.716  0.660 | 1.060  0.518  0.714 | 3-Cal  2-Cal, 1-Val  2-Cal, 1-Val |
